# The impact of fungicide treatments on yeast biota of Verdicchio and Montepulciano grape varieties

**DOI:** 10.1371/journal.pone.0217385

**Published:** 2019-06-20

**Authors:** Alice Agarbati, Laura Canonico, Maurizio Ciani, Francesca Comitini

**Affiliations:** Dipartimento di Scienze della Vita e dell’Ambiente, Università Politecnica delle Marche, Ancona, Italy; Universidade do Minho, PORTUGAL

## Abstract

Yeast species that colonize the surface of grape berries at harvest time play an important role during the winemaking process. In this study, the use of culturable microbial techniques permitted a quantitative and qualitative inventory of the different yeast species present on the grape berry surfaces of Montepulciano and Verdicchio varieties when treated with conventional and organic fungicides. The results show that the most widespread yeast species at harvest time were *Aureobasidium pullulans* and *Hanseniaspora uvarum*, which are considered normal resident species and independent of the grape varieties and treatments applied. Specific differences when comparing the grape varieties were observed in species and were detected at a lower frequency; *Pichia* spp. were prevalent in Verdicchio, whereas *Lachancea thermotolerans* and *Zygoascus meyerae* were found in Montepulciano. In both vineyards, the farming treatments improved the competitiveness of *A*. *pullulans*, which was probably due to its reduced susceptibility to treatments that improved the competition toward other fungi. In contrast, the fermenting yeast *H*. *uvarum* was negatively affected by fungicide treatments and showed a reduced presence if compared with untreated grapes. Organic treatments directly impacted the occurrence of *Issachenkia terricola* in Montepulciano grapes and *Debaryomyces hansenii* and *Pichia membranifaciens* in Verdicchio. Conversely, a negative effect of organic treatments was found toward *Metschnikowia pulcherrima* and *Starmerella bacillaris*. Overall, the data suggest that the yeast community colonizing the grape berry surface was influenced by both grape variety and farming treatments, which characterized the yeast biota of spontaneous must fermentation.

## Introduction

Grapes represent a complex ecological niche where filamentous fungi, yeasts and bacteria cohabitate. The microbiome includes species at a concentration that mainly depends on the grape ripening stage and the availability of nutrients. However, the microbial communities of grapes may be affected by many other variables, such as pedoclimatic factors, viticultural practices, diseases and pests that could modify grape integrity [1]. In general, the yeast populations of mature grapes are comprised by 10^3^ and 10^5^ cells/g, but higher values (approximately one log) have also been found on damaged berries where the availability of sugar and nutrients is higher [2].

Among biotic factors, microbial vectors, such as bees and wasps, can actively transfer yeasts to the grape surfaces [3–5] where it can establish synergistic or antagonistic behaviors between various genera and species of bacteria, yeasts and molds that cohabit together. The microbiome composition and complexity also depend on the interactions between individuals, and the resulting consortium is generally stable over time. Relative to abiotic factors, the climatic and microclimatic conditions, including the effect of temperature, UV exposure, rainfall, sunlight and winds, can influence microbial populations. However, the results are often unclear because it is not easy to apply the scientific method to the function of natural events. For instance, rainy vintages lead to higher use of phytochemicals and show higher fungal proliferation and higher berry damage in conjunction with lower UV irradiation [6].

Concerning the total yeast counts, Combina et al. [7] found that rainy years increased yeast presence. This climatic condition probably increases the berry volume and permits the release of juice in joint areas, such as the part between the pedicel and the berry, and higher exosmosis leads to nutrients on the grape surface. With careful and sound berry sampling, Čadež et al. [8] found that colder harvests with higher rainfall lead to increased yeast counts. In contrast, Comitini and Ciani [9] found 10-fold less total counts in years with high rainfall. In addition, the geographic location, grape variety and vineyard age and size can influence the composition and occurrence of microflora that are present on the surface of grape berries.

Another important aspect is related to vineyard treatments. Viviani-Nauer et al. [10] found that pesticides decreased the yeast population and diversity in fermenting musts, whereas Cabras et al. [11] reported the absence of an effect on the fermentation activity of *Saccharomyces cerevisiae* by different fungicides and a stimulation of fermentation by *Kloeckera apiculata* was observed. Ganga and Martínez [12] detected less diversity of non-*Saccharomyce*s species in association with the systemic use of chemical fungicides against *Botrytis cinerea*.

Čadež et al. [8], with careful berry selection, showed that after the safety interval, fungicides against *B*. *cinerea* had a minor impact on the composition of grape berry microbiota and untreated grapes were less contaminated. Recent works concerning the differences between organic and conventional farming systems concluded that organic farming leads to higher biodiversity both in *S*. *cerevisiae* and in non-*Saccharomyces* yeasts [13–16].

In this study, the yeast culturable biota of the grape surface of two Italian varieties was monitored at harvest time and during the spontaneous fermentations of grape samples when conducted under sterile conditions using conventional culture methods. The influence on the yeast community of conventional and organic treatments was also evaluated by comparing the samples with untreated grapes.

## Materials and methods

### Viticultural habitats and grape sampling

The grapes used in this study were obtained from two vineyards of two typical grape varieties of the Marche region, in the center of Italy: Verdicchio and Montepulciano. In particular, the Verdicchio vineyard is located in the Montecarotto locality (43°31’41”N, 13°03’59”E; 334 m altitude) within the *Denominazione di Origine Controllata* (D.O.C.), and the main climatic condition in September (sampling period) was 18.7°C for air temperature, had 50.4% humidity and included 9 rainy days. Montepulciano vineyard is located in the Sirolo locality (43°31’20N, 13°36’53”E; 97 m altitude), and the main climatic condition in October (sampling period) was 14.9°C for air temperature, had 82% humidity and included 15 rainy days. Both vineyards have employed three different management systems: organic, conventional and with no treatment. To exclude any cross-contamination between different treatments, the minimum distance between each block of rows was approximately one kilometer from each other for all of the grapes; and within the same vineyard, the grapes are exposed to the same slope, sun, and shade and have similar soil characteristics. The harvest was carried out, in both varieties, at full ripeness (15 September for Verdicchio; 10 October for Montepulciano).

In the organic treatment (both varieties: Montepulciano and Verdicchio), a Bordeaux mixture (20 g/L of copper (II) sulfate + 13 g/L of calcium hydroxide with pH 6.6) and sulfur (Microthiol disperss, UPL EUROPE Ltd., Warrington WA3 6YN, Great Britain) were used. For both vineyards, 15 consecutive treatments were performed from April 20^th^, 2016, to August 17^th^, 2016.

In the conventional Verdicchio treatment, viticulture commonly included chemical compounds with fungicide activity and were employed as follows: copper-oxychloride (Coprantol WG, Sygenta Italia Spa, Casalmorano, Cremona, Italy) (1), sulfur (Tiovit jet, Sygenta Italia Spa, Casalmorano, Cremona, Italy) (1), cyclohexanol + 1,2- propanediol + abamectin + 2,6-di-terbutylp-cresol (Vertimec 1.9 ec, Sygenta Italia Spa, Casalmorano, Cremona, Italy) (1), iprovalicarb + technical copper oxychloride (Melody compact WG, Bayer Crop Science, Monheim am Rein, Germany) (1), sulfur (Selenium free) + terpene alcohols + sodium salt of an aromatic polymer (Heliosulfure S, Biogard, Cesena, Italy) (12), a Bordeaux mixture (11), coppery sulfur (1), and phosphorus pentoxide + potassium oxide (Landamine PK, BMS Micro-Nutrients N.V., Bornem, Belgium) (1). Twelve consecutive treatments were performed from April 18^th^, 2016, to August 12^th^, 2016.

In the conventional Montepulciano treatment, viticulture commonly included chemical compounds with fungicide activity and were employed as follows: spiroxamina (Prospher300 CS, Bayer Crop Science, Monheim am Rein, Germany), copper-oxychloride (Coprantol, Sygenta Italia Spa, Casalmorano, Cremona, Italy), sulfur (Tiovit jet, Sygenta Italia Spa, Casalmorano, Cremona, Italy), fosetyl-Al+copper sulfate (R6 Erresei Bordeaux WG, Bayer Crop Science, Monheim am Rein, Germany), Metalaxyl-M14+ copper-oxychloride (RidomilGold, Sygenta Italia Spa, Casalmorano, Cremona, Italy), quinoxyfen+myclobutanil+coformulants (Arius System Plus, Dow AgroSciences, Indianapolis, Indiana, USA), copper sulfate and sulfur. Nine consecutive treatments were performed from March 10^th^, 2016, to July 17^th^, 2016.

During the harvest period, several grape samplings were performed using sterile plastic bags. Each sample consisted of an undamaged ripe grape bunch (approximately 1 kg per bunch). In total, 50 samples were collected: ten samples of organic and conventional Montepulciano grapes; five samples of non-treated Montepulciano grapes; thirteen samples of organic Verdicchio grapes; ten samples of conventional Verdicchio grapes and two samples of no-treated Verdicchio grapes. All of the samples were immediately transported to the laboratory on ice for processing.

### Spontaneous fermentations

The grapes were placed into sterile bags and were hand-crushed and shaken at 120 rpm for 30 minutes on an MAXQ 4450 (Thermo Fisher Scientific, Waltham, Massachusetts, USA). One milliliter of each fresh must was collected and used for yeast isolation and enumeration. The remaining grape juice with skins was transferred into 250 mL sterile Erlenmeyer flasks, closed with Pasteur bungs (to allow CO_2_ to escape from the system) and set up for spontaneous fermentation at 25°C under static conditions. After 7 and 15 days from the start to the spontaneous fermentation, the samples were collected to evaluate the yeast population by viable cell counts.

### Yeast enumeration and isolation

Samples from fresh musts and during fermentation were collected and used for monitoring the yeast populations at the beginning and after 7 and 15 days of fermentation. For total yeast enumeration, serial decimal dilutions in sterile water were prepared and then spread on Wallerstein (WL) nutrient agar (Merck KGaA, Germany) supplemented with 0.005% chloramphenicol (Thermo Fisher GmbH, Germany) and 0.02% biphenyl (Sigma-Aldrich, <Saint Louis, Missouri, USA) to suppress the bacteria and reduce the growth of molds, respectively. The plates were incubated at 25°C for four days. After macro- and micro-morphological analysis and in proportion to their frequencies, the yeast isolation was conducted on plates that contained between 100 and 300 colonies. Approximately 10% of the colonies per plate were isolated on YPD agar (1% Yeast Extract, 2% Peptone, 2% D-glucose, and 2% Agar) from each sample and at each time of sampling [17,18]. The total isolates were 1,240. The yeast strains were preserved in 40% (v/v) glycerol at -80°C.

### DNA extraction and yeast identification

The 1,240 isolated strains, that showed identical macro- and micro-morphological characteristics, were grouped and representative isolates were used for genomic DNA analysis. DNA was extracted according to the method described by Stringini et al. [19]. Using primer set ITS1 (5’-TCCGTAGGTGAACCTCGCG-3’) and ITS4 (5’-TCCTCCGCTTTATTGATATGC-3’) [20], the ITS1-5.8S rRNA-ITS2 region was amplified by PCR. The PCR was performed as described by Esteve-Zarzoso and co-workers [21]. The PCR products were separated in 1.5% (w/v) agarose gel (stained with ethidium bromide) using 0.5x TBE buffer by horizontal electrophoresis (Bio-Rad, Hercules, USA). The identities of the representative yeasts were obtained by sequencing. The BLAST program [22] and the GenBank database (http://www.ncbi.nlm.nih.gov/BLAST) were used to compare the sequences provided with those already in the data library. The inclusion of obtained sequences into the NCBI GenBank data library has been completed under the accession numbers from MK351988 to MK352096.

### Statistical analysis

The relative abundance of species was obtained by calculating the corresponding portion of each species with respect to the total yeast detected in the samples and based on the colony counts. The analysis of variance was conducted using the JMP 11 from SAS program. Furthermore, the results obtained from the analysis of microbial diversity on the grape surface of different vineyards employing different agronomic practices and yeast dynamics during spontaneous fermentation were examined with Unscrambler 7.5 software (CAMO ASA, Oslo, Norway) to obtain the Principal component analysis (PCA).

### Ethics statement

For this research is not require an ethics statement, and the authors confirm that the field studies did not involve endangered or protected species.

## Results

### Effect of grape variety on the yeast community at harvest time

**Fig 1:**
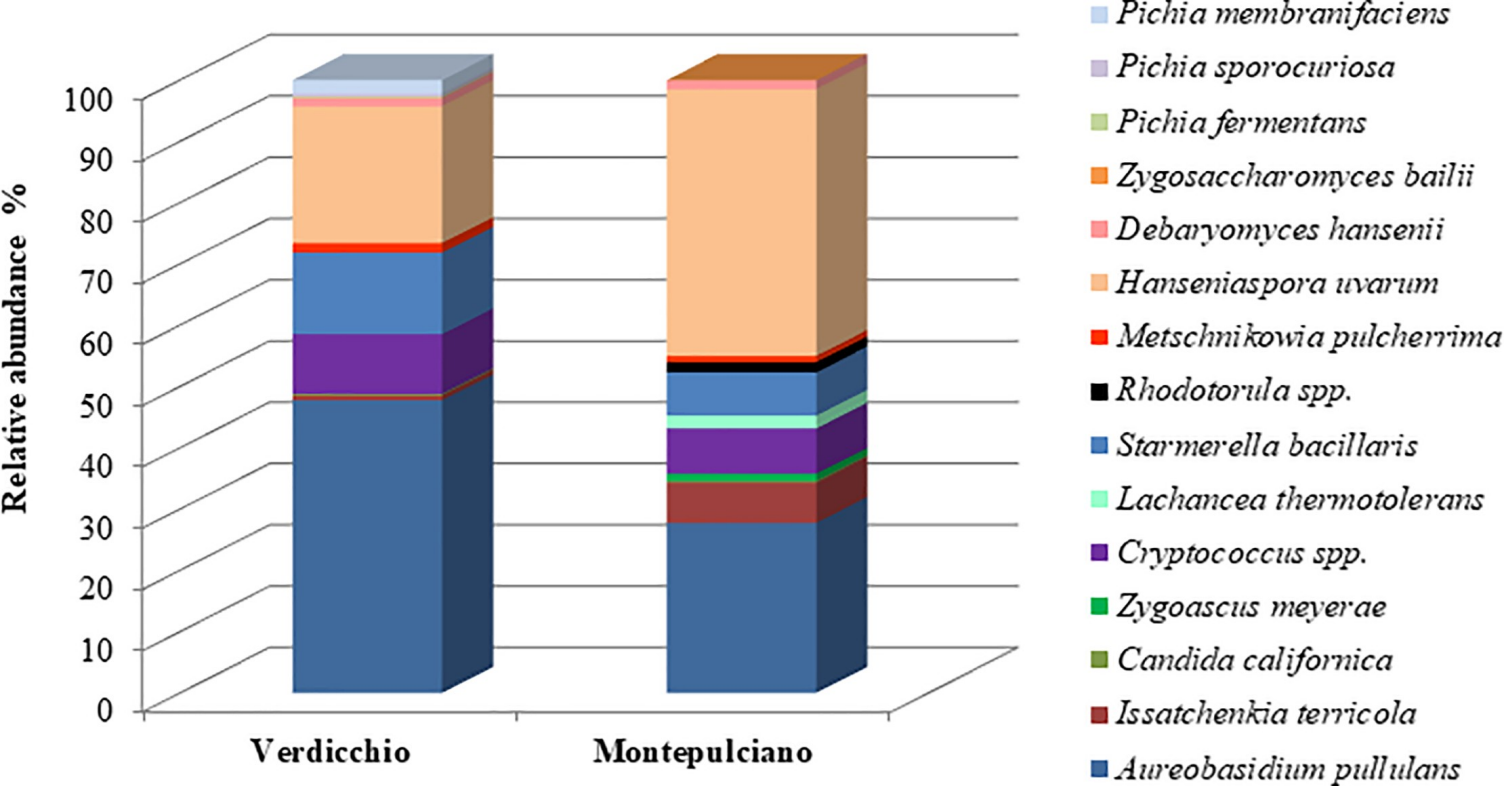
Mean values (%) of the initial yeast community in Verdicchio and Montepulciano grapes.

Microbial community associated with the grape surface of the Verdicchio and Montepulciano varieties was evaluated. From the general framework (Fig 1), it was observed that both grape varieties presented an abundance of yeasts, such as *Aureobasidium pullulans* and *Cryptococcus* spp., with oxidative metabolism. Together, these represent 60% and 40% of the total yeasts present on grape berries of Verdicchio and Montepulciano, respectively. In particular, out of the 60% associated with Verdicchio grapes, 50% belong to *A*. *pullulans* and 10% to *Cryprococcus* spp. The 40% of yeasts with oxidative metabolism associated with Montepulciano were 30% *A*. *pullulans*, 7% *Cryptococcus* spp. and 3% minor representative species. In terms of relative abundance and among the fermenting yeasts, *Hanseniaspora uvarum* (22% Verdicchio grapes and 43% Montepulciano grapes) and *Starmerella bacillaris* (13% Verdicchio grapes and 7% Montepulciano grapes) were the most abundant and constantly present species found in both varieties. The Montepulciano variety was characterized by a consistent presence of *Issatchenkia terricola* (7%). Both grape varieties showed the presence of weak fermenting yeasts such as *Metschnikowia pulcherrima* and *Debaryomyces hansenii* (1.0–1.5%) and *Candida californica* (<1%). At low relative abundance (1.0–2.0%), some yeast species were found in one or the other grape varieties. *Pichia sporocuriosa*, *Pichia fermentans* and *Pichia membranifaciens* were found only in the Verdicchio grape variety, whereas species such as *Lachancea thermotolerans*, *Zygoascus meyerae* and *Rhodotorula* spp. were only found in grapes from the Montepulciano variety. In summary, no substantial differences were found among the two grape varieties regarding the main yeast species (oxidative and low fermenting species) that colonized the grape berry surface. A statistically significant difference was found for the relative abundance of *H*. *uvarum* more present on Montepulciano grape variety. This difference, in addition to the variety effect, could also due to other intrinsic variables of their cultivation management such as the different harvest time of grapes (Table A in S1 File). On the other hand, differences were detected in species found at a low frequency (often fermenting species) that were only isolated in one or another grape variety.

### The influence of fungicide treatments on the yeast community at harvest time

**Fig 2:**
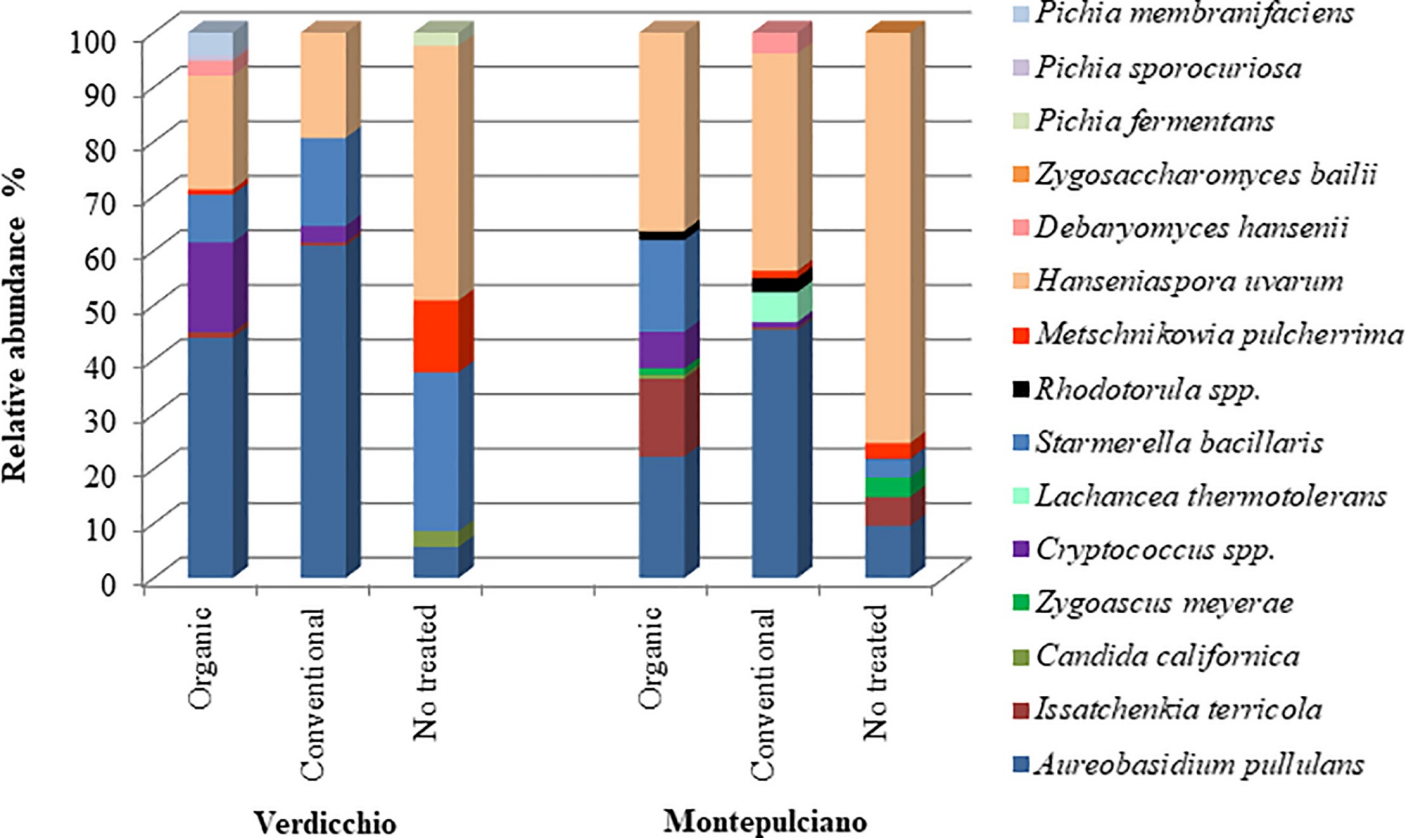
The average percentages of yeast species detected in organic, conventional and non-treated samples of Verdicchio and Montepulciano grapes at harvest time.

The yeast community that colonizes grape surfaces was analyzed for the influence of the fungicide treatments. Both Verdicchio and Montepulciano varieties have been subjected to an organic and a conventional treatment. The results showed that in Verdicchio samples (Fig 2), the yeast-like *A*. *pullulans* was favorited by fungicide treatments in comparison to untreated samples (t = 0.05) probably due to the lower competition of the yeasts affected by treatments. Indeed, in both organic and conventional samples, this yeast was the most abundant species (44% and 60%, respectively) while in untreated grapes it was only 5% of the whole yeast population. The same behavior was observed in Montepulciano samples (22%, 45% and 10% in organic, conventional and untreated samples, respectively) (Fig 2). Conventional treatments seem to exert more selective pressure on the yeast community and favor the colonization of *A*. *pullulans* (around half of the yeast community). Similar to *A*. *pullulans*, the occurrence of *Cryptococcus* spp. seemed to be positively influenced by the treatments (and this was absent in the untreated grapes). Different from *A*. *pullulans*, the organic treatments positively affected the colonization of *Cryptococcus* spp. in comparison with conventional treatments in both Verdicchio and Montepulciano varieties even if only in Verdicchio variety a significant difference was found (Table B in S1 File) (16% and 3% in organic and conventional Verdicchio grapes, respectively, and 7% and 1% in organic and conventional Montepulciano grapes, respectively).

*H*. *uvarum* was the second most abundant species in the treated grapes of both varieties. This apiculate yeast did not seem to be influenced by the type of treatment (26% and 20% in organic and conventional Verdicchio grapes and 36% and 40% in organic and conventional Montepulciano grapes, respectively). In untreated grapes, *H*. *uvarum* was the most abundant species (47% and 75% in Verdicchio and Montepulciano varieties, respectively) and showed significant differences in comparison with both treated grapes (organic and conventional) but only in Montepulciano variety (Table C in S1 File).

*S*. *bacillaris* species showed a wide variability among the treatments/varieties. In the Verdicchio variety, *S*. *bacillaris* decreased in the treated grapes (9% in organic samples and 16% in conventional samples) compared to the untreated ones (29%) Fig 2). In Montepulciano, *S*. *bacillaris* was more present in organic grapes (17%), absent in conventional samples and poorly present in untreated samples (4%).

Organic treatments favorably affected the presence of *I*. *terricola* and showed an abundance of 14% in organic grapes and only 5% in untreated grapes, and *I*. *terricola* was almost absent in conventional samples of the Montepulciano variety while it was present at a very low relative abundance (<1%) in the Verdicchio variety. A positive effect of organic treatments was also exerted on some low abundance species only present in the Verdicchio variety, such as *P*. *membranifaciens* (4%) and *P*. *sporocuriosa* (1%), that were only found in the grapes treated with copper and sulfur. Differently, a negative effect of organic treatments was shown toward *M*. *pulcherrima*. Indeed, this yeast species was significantly present in untreated (13 and 3% in Verdicchio and Montepulciano grapes, respectively) and poorly present or absent in treated grapes (Tables B and C in S1 File). *D*. *hansenii* was detected only in treated samples, while *P*. *fermentans* and *C*. *californica* were generally found in untreated grapes (2.5% and 3%, respectively). *Rhodotorula* spp., a ubiquitous yeast, was present only after the treatments (1.67% and 2.57% in Montepulciano organic and conventional samples, respectively). Fermenting yeasts detected only in the Montepulciano grape variety showed a different response toward fungicide treatments. *L*. *thermotolerans* was only present in conventional treatment samples (5.5%), whereas *Z*. *meyerae* and *Zygosaccharomyces bailii* were mainly detected in untreated samples and almost absent in conventional grapes.

### Yeasts dynamics during spontaneous fermentation

#### Middle fermentation

**Fig 3:**
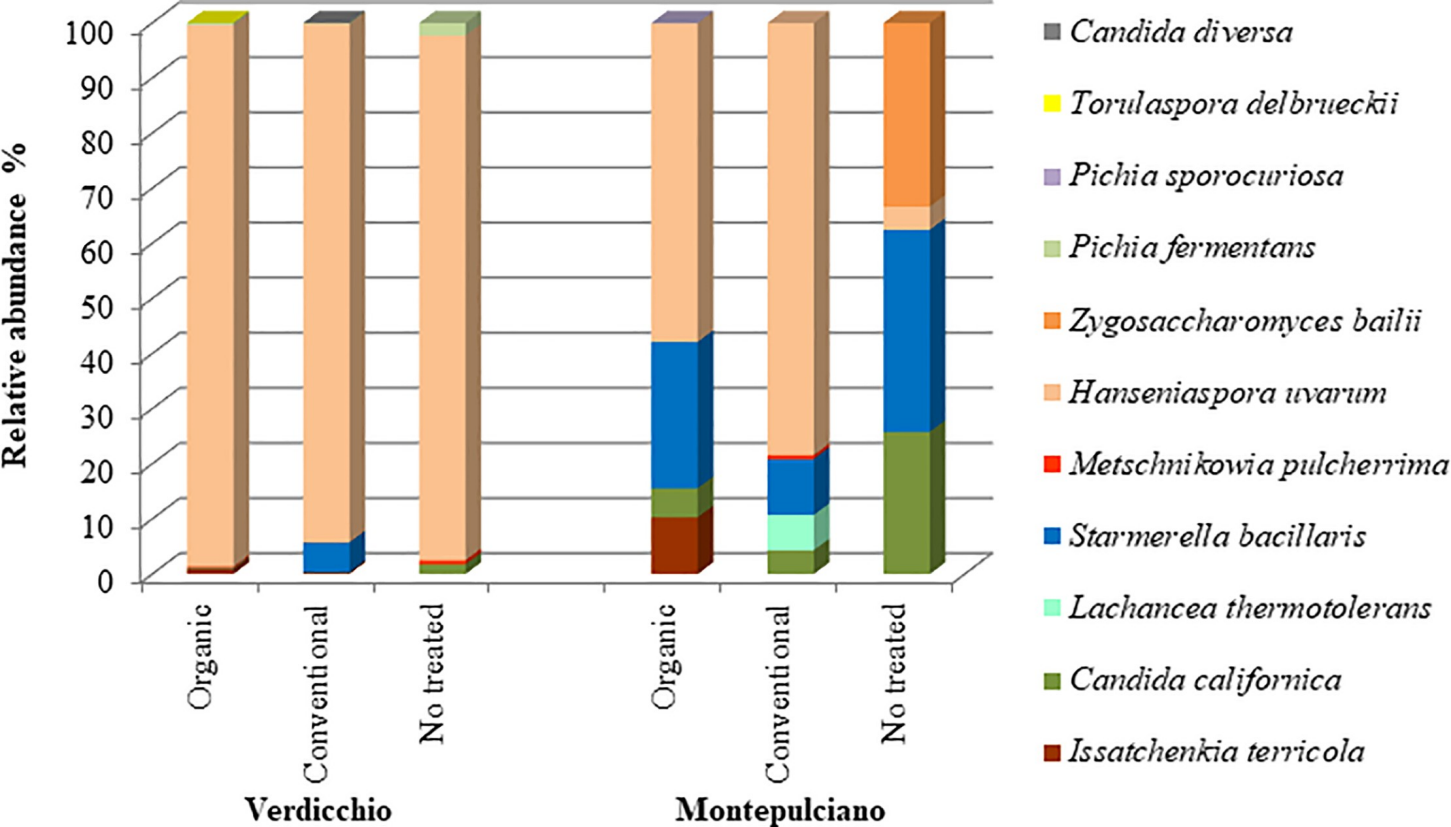
Average percentages of yeast species detected in organic, conventional and non-treated samples of Verdicchio and Montepulciano samples after 7 days of fermentation.

The microbial community evolution during spontaneous fermentation was monitored through viable counts after 7 and 15 days from the start of fermentation. After 7 days (approximately middle fermentation) the yeast population increased by approximately two log (from 8.0×10^5^ CFU/ml to 6.0×10^7^ CFU/ml) in Verdicchio samples and only one log in Montepulciano samples (from 1.7×10^6^ CFU/ml to 1.5×10^7^ CFU/ml) (Tables D and E in S1 File). As expected, the environmental conditions determined a selection in favor of fermenting yeasts. Indeed, the oxidative yeasts *A*. *pullulans* and *Cryptococcus* spp. disappeared from all of the samples. *H*. *uvarum*, which was already well represented at the beginning of fermentation (22%), became the dominant species in all of the Verdicchio trials (Fig 3) and the only occasionally fermenting yeast species (*I*. *terricola*, *S*. *bacillaris*, *C*. *californica*, *M*. *pulcherrima*, *P*. *fermentans*, *Torulaspora delbreuckii*, and *Candida diversa*) were found at low relative abundance (all together at approximately 4%). This picture is nearly confirmed in Montepulciano treated samples where other yeast species participated in the fermentation process. In untreated samples, *H*. *uvarum* was present at only 4% of the total yeast population, but other fermenting species *S*. *bacillaris* (37%), *Z*. *bailii* (34%) and *C*. *californica* (26%) appeared. *S*. *bacillaris* and *C*. *californica* were present in all Montepulciano samples even if they were more abundant in untreated samples. Montepulciano samples can be differently recognized by a relevant presence of: i) *I*. *terricola* in organic samples, ii) *L*. *thermotolerans* in conventional samples, and iii) *Z*. *bailii* in untreated samples (Fig 3).

#### End of fermentation

**Fig 4:**
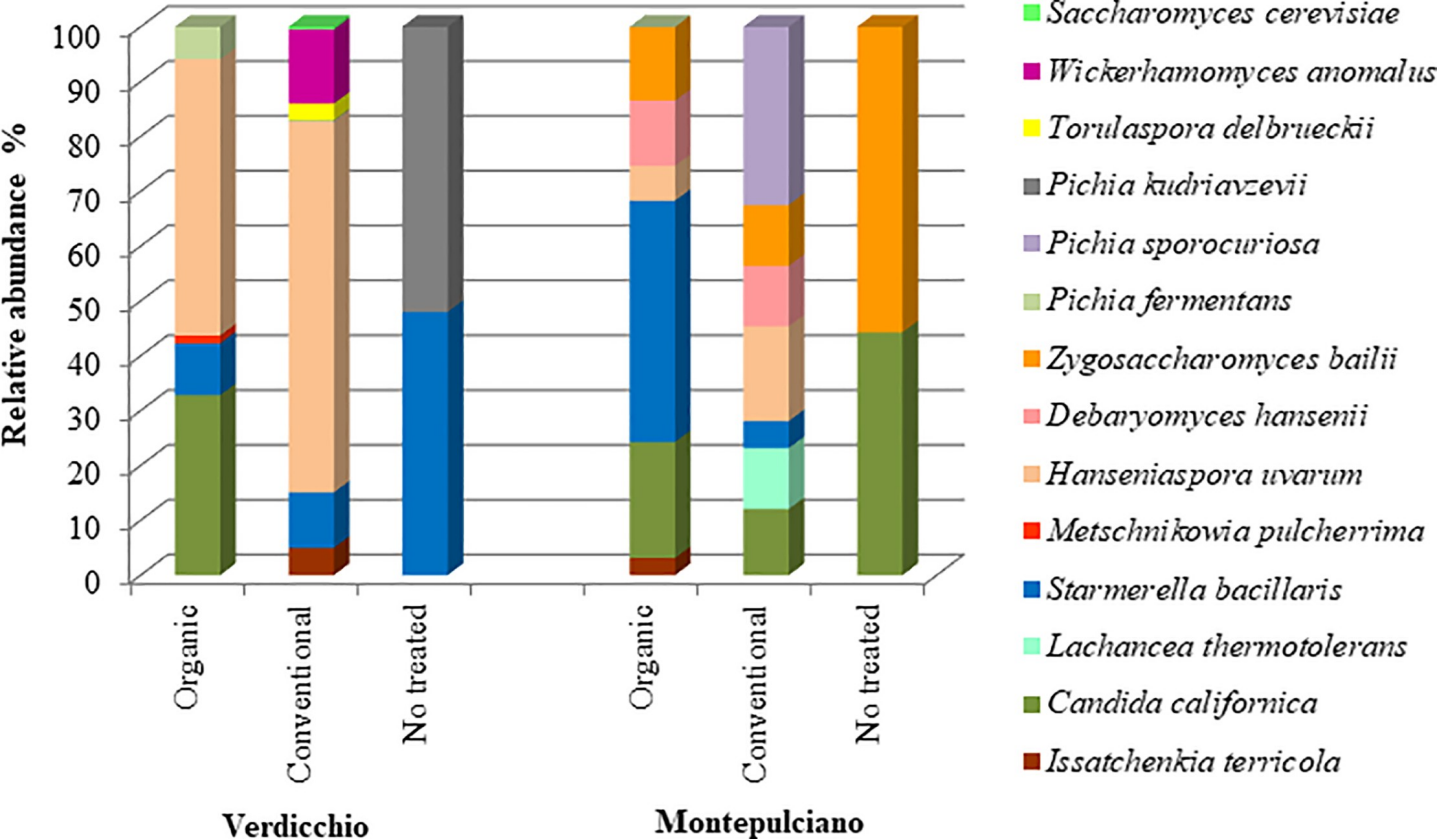
Average percentages of yeast species detected in organic, conventional and non-treated samples of Verdicchio and Montepulciano samples after 15 days of fermentation.

The results of the microbiological analysis conducted after 15 days of spontaneous fermentation are shown in Fig 4. Due to the reduced size of each grape juice sample, the presence and participation of the fermentation process of the strongest fermenting yeast, *S*. *cerevisiae* and *T*. *delbrueckii*, were very limited (<1% and 3%, respectively, and only in Verdicchio conventional samples). *H*. *uvarum* remained the dominant species in organic and conventional Verdicchio samples (50% and 67.5%, respectively), while it showed a significant reduced presence in Montepulciano treated samples (7% in organic and 17% in conventional samples). In organic samples, *C*. *californica* (33% and 21% in Verdicchio and Montepulciano samples, respectively) and *S*. *bacillaris* (10% and 44% in Verdicchio and Montepulciano samples, respectively) were the other dominant species. The other yeast species were *P*. *fermentans* (6%) in Verdicchio samples and *Z*. *bailii* (13%), *D*. *hansenii* (12%) and *I*. *terricola* (3%) in Montepulciano samples. In conventional samples other than *H*. *uvarum*, the species were *W*. *anomalus* (13%), *S*. *bacillaris* (10%) and *I*. *terricola* (5%) in Verdicchio varieties and *P*. *sporocuriosa* (32%), *C*. *californica* (12%), *L*. *thermotolerans* (11%), *D*. *hansenii* (11%), *Z*. *bailii* (11%) and *S*. *bacillaris* (5%) in Montepulciano samples. In untreated samples, *H*. *uvarum* was not found in either variety where the species was detected: *Pichia kudiavzevii* (52%) and *S*. *bacillaris* (48%) in the Verdicchio variety and *Z*. *bailii* (56%) and *C*. *californica* (44%) in the Montepulciano variety.

### Principal component analysis (PCA) of the yeast community

**Fig 5:**
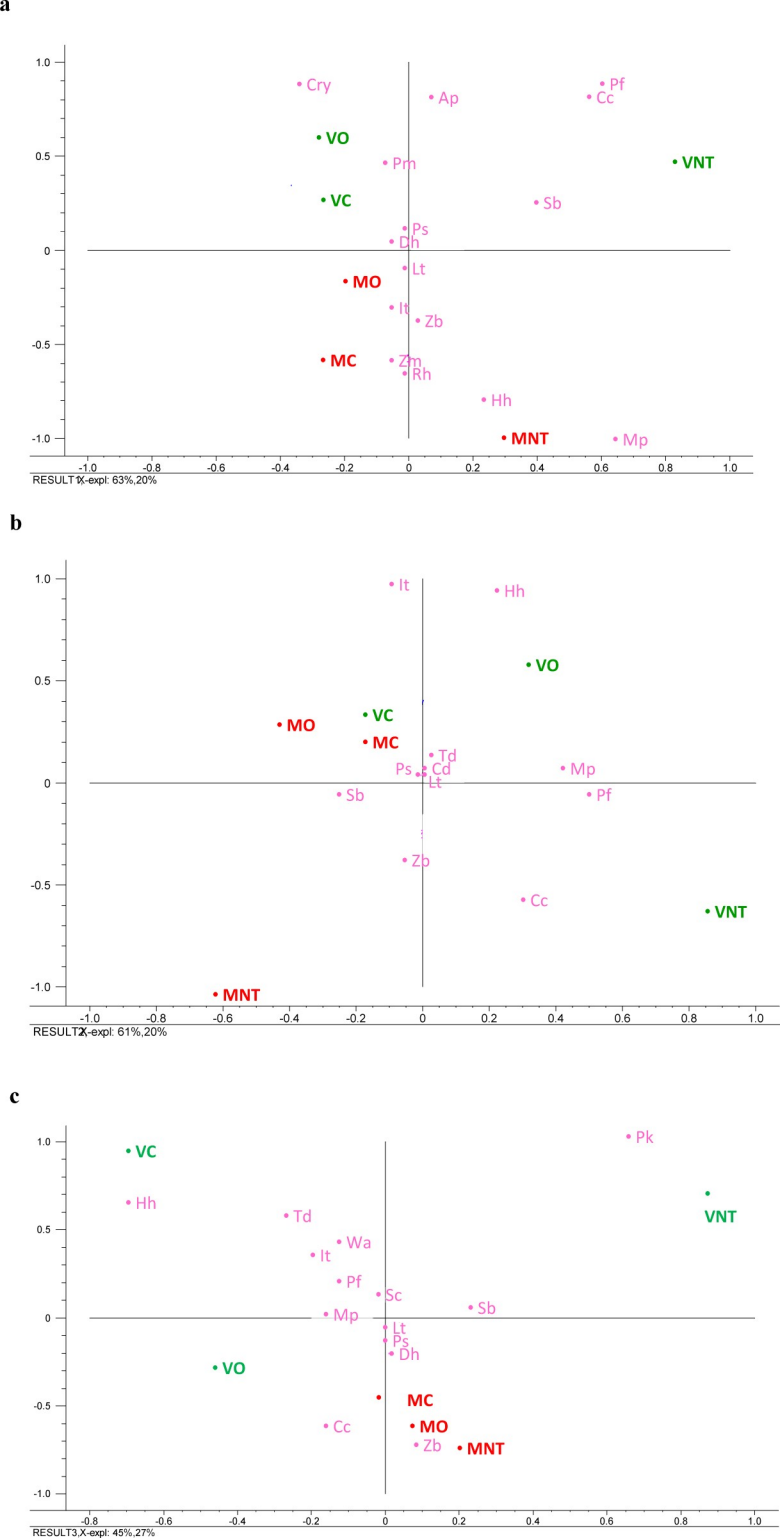
Principal component analysis related to the yeast community of samples coming from Verdicchio (V) and Montepulciano (M) vineyards subjected to organic (O) and conventional (C) fungicide and non-treated (NT) treatments. (a) The yeast community on the grape surface detected at harvest time; (b) the yeast community of samples at 7 days of spontaneous fermentation; and (c) the yeast community of the samples at 15 days of spontaneous fermentation. *A*. *pullulans* (Ap); *I*. *terricola* (It); *C*. *californica* (Cc); *Z*. *meyerae* (Zm); *Cryptococcus* spp. (Cry); *L*. *termotolerans* (Lt); *S*. *bacillaris* (Sb); *Rhodotorula* spp. (Rh); *M*. *pulcherrima* (Mp); *H*. *uvarum* (Hu); *D*. *hansenii* (Dh); *Z*. *bailii* (Zb); *P*. *fermentans* (Pf); *P*. *sporocuriosa* (Ps); *P*. *membranifaciens* (Pm); *T*. *delbrueckii* (Td); *C*. *diversa* (Cd); *P*. *kudriavzevii* (Pk); *W*. *anomanuls* (Wa); *S*. *cerevisiae* (Sc).

The PCA of the overall yeast microbiome of the grape berry surface at the harvest period and during a spontaneous fermentation revealed a yeast population diversity among the samples of grapes subjected to organic and conventional fungicide practices and untreated samples coming from two different varieties/vineyards (Fig 5). The biplots were obtained by evaluating the relative yeast species abundance, the grape varieties and the fungicide treatments.

At the harvest time, PC1 (63%) showed a differentiation between untreated (right quadrants) and treated samples independently by the type of treatment while PC2 (20%) distinguished between Verdicchio (upper quadrants) and Montepulciano (lower quadrants) samples (Fig 5A). These data suggest an evident impact of fungicide treatments on yeast biota associated with the grape berry surface. Moreover, the grape varieties showed a different yeasts colonization, although different harvest times (with consequent different climatic conditions) and agronomic management could contribute to this yeast differentiation. In this regard, *M*. *pulcherrima* and *H*. *uvarum* in Montepulciano and *P*. *fermentans* and *C*. *californica* in Verdicchio varieties were the main characterizing species of untreated samples, whereas oxidative yeasts species mainly differentiated the treated samples. The spatial distribution of PCA confirmed that the species that linked the two grape varieties were: *Z*. *meyerae*, *I*. *terricola*, *Rhodotorula* spp., and *Z*. *bailii* for Montepulciano and *P*. *membranifaciens*, *P*. *sporocuriosa* and *P*. *fermentans* for Verdicchio.

At 7 days of fermentation (Fig 5B), a general reduction and simplification of the yeast community was observed. However, the untreated samples remained separated from the treated samples (down and upper quadrants, respectively). PC1 (61% of variance explained) distinguished Verdicchio samples from those of Montepulciano even if conventional samples were more closely related than organic ones.

At the end of fermentation (Fig 5C), all of the Montepulciano samples were grouped in the right/lower quadrant and mainly characterized by *C*. *californica* and *Z*. *bailii*. Furthermore, *P*. *sporocuriosa* characterized Montepulciano conventional samples and *S*. *bacillaris* characterized Montepulciano organic samples. In contrast, all of the Verdicchio samples were differently distributed in the graphic space and indicated remarkable differences for the relevant presence of *P*. *kudriavzevii* (untreated samples), *H*. *uvarum* and *T*. *delbrueckii* (conventional samples), and *C*. *californica* organic samples).

## Discussion

In recent years, the investigation of the geographic distribution of the microbial community of wine grapes revealed a geographic delineation of the yeast communities conditioned by several factors such as cultivar, vintage, climate and agricultural practices [23–26].

The influence of farming practices used in the vineyard and on the yeast, biota associated with the grape berry surface was recently investigated [18, 27–29]. In the present study, the impact of organic and conventional treatments on the occurrence of yeast species in two Italian varieties was evaluated. The total yeasts recovered at harvest time were from 10^5^ to 10^6^ CFU/ml and in accordance with the yeast presence in grapes described in previous studies [7,30].

*A*. *pullulans* was the first and most abundant species found in both treated Verdicchio samples and in conventional Montepulciano grapes and in agreement with results obtained from Setati et al. [29]. In organic Montepulciano samples, *A*. *pullulans* represented the second most abundant species found, and this disagrees the results obtained by Renouf et al. [30] that did not find *A*. *pullulans* on the grape surface. These yeasts seem to be favorably affected by farming treatments, which is probably due to their improved competitiveness towards other fungi in the presence of fungicides and their capacity to detoxify CuSO_4_ as reported by Schmid et al. [31]. Their results support the key role of the yeast-like fungus *A*. *pullulans* in explaining the functional differences between organic and conventional agricultural systems. Indeed, it has long been known that *Aureobasidium* can utilize inorganic sulfur and is able to absorb, and in this way detoxify, copper [32–33]. In untreated grapes, *A*. *pullulans* represents only a minor component of the whole yeast population.

*H*. *uvarum* was the most abundant fermenting species in both varieties, although the fungicide treatments significantly reduced its presence. These results, which confirmed previous results, are in accordance with the current literature reviewed by Pretorius [34].

A negative effect of both conventional and organic treatments was detected toward *M*. *pulcherrima* since a significant decrease in treated samples was found. The same results were described by Milanović and co-workers [18] but only in organic samples. This finding highlighted that *M*. *pulcherrima*, antagonistic and antimicrobial yeast [35–38], is negatively influenced by fungicide treatments and particularly organic ones. Considering that this yeast species revealed a positive contribution to the analytical and aromatic composition and complexity of wine [39–41], fungicide treatments may reduce this yeast’s positive contribution to fermentation and wine composition.

The monitoring of fermentation conducted at the laboratory scale elucidated the relationship between yeast occurrence on the grape surface and the potential influence during applicative wine management.

According to Bagheri et al. [42], our results clearly showed a decline at the start of fermentation of the oxidative strains; this decline was probably due to the anaerobic conditions created by the fermentation process. As expected, in the middle of fermentation, *H*. *uvarum* became the most representative species in all of the samples [18]. At this stage, *P*. *fermentans* and *C*. *californica* in Verdicchio and Z. *meyerae* and *Z*. *bailii* in Montepulciano seems to characterize the yeast biota of the two varieties. Regarding the fermenting yeast *L*. *thermotolerans*, the results obtained seem to highlight the favorable effect of conventional treatments on this species that was only found in Montepulciano. Cordero-Bueso et al. [13] described *L*. *thermotolerans* as the predominant non-*Saccharomyces* species found in both organic and conventional samples without relevant differences between the treatments. In our study, this yeast was present on the grape berry surface at harvest time and survived until the end of spontaneous fermentation. Its initial concentration probably plays a significant role in establishing yeast-yeast interaction that allows itself to compete and survive during fermentation [42]. In contrast to *L*. *thermotolerans*, *Z*. *bailii* was found in all of the samples of the Montepulciano varieties; this outcome indicated that this species was not affected by organic or conventional treatments.

In Verdicchio samples, the fermenting yeasts, *W*. *anomalus* and *T*. *delbrueckii*, seems to characterize the conventional samples. The presence of *T*. *delbrueckii* agrees with the results of Cordero-Bueso et al. [13] that detected this species in Barbera musts coming from conventional grapes. *T*. *delbrueckii* was found at a low frequency only at the end of fermentation as reported by Pinto et al. [43], whereas Bagheri et al. [42] found *T*. *delbrueckii* only in samples coming from an integrated vineyard. However, *W*. *anomalus* represented the second most abundant species probably due to its capacity to persist at the typical end-fermentation conditions [44–45]. Indeed, it established its ability to tolerate up to a 12% ethanol concentration and to produce a killer toxin to compete against other yeasts [46–47].

The strong fermenting yeast *S*. *cerevisiae* was poorly detected and emerged at the end of fermentation and only in Verdicchio conventional samples. These data confirm that the best fermenting yeasts is very poorly present on the grape berry surface at harvest time [17, 48]. On the other hand, fermenting yeasts present at low frequencies at harvest time, took over the spontaneous fermentation differently depending on the fungicide treatments. This is the case of *C*. *californica* in organic samples of Verdicchio variety and *S*. *bacillaris* in organic samples of Montepulciano variety (both species absent or present at very low frequencies in the conventional samples). Differences in the dominant yeast species at the end of fermentation in the grape variety were also found (high presence of Z. *bailii i*n Montepulciano and *H*.*uvarum* in Verdicchio) and could be due to the overall differences between the varieties (characteristic of grapes, time of harvest and agronomic management).

Overall, the data suggest that the yeast community colonizing the grape berry surface was influenced by agricultural treatments. *A*. *pullulans* and *H*. *uvarum* were the dominant yeast species at harvest time even if their relative frequencies were strongly influenced by fungicide treatments. Fermenting yeast species differently colonized the grape surface and characterized microfermentations trials of Verdicchio and Montepulciano varieties. These fermenting yeast population changes from varieties are conditioned by pesticide treatments and could be expected to have some impact on the fermentation process and wine composition and their evaluation should receive further attention.

## Supporting information

S1 File Supplemental Materials. (Table A) Analysis of variance (ANOVA) of Verdicchio and Montepulciano samples at harvest time. The significant differences were determined using t-Test, and the data were considered significant if the associated P values was <0.05. Data with different letters (A, B) within each row are significantly different. (Table B) Analysis of variance (ANOVA) of Verdicchio samples at harvest time and after 7 and 15 days of spontaneous fermentation. Letters O, C and NT indicated organic, conventional and untreated farming management, respectively. For each yeast species detected and for each sampling time, the different letters (A, B) indicated significant differences between the samples (p < 0.05) using t-Test. (Table C) Analysis of variance (ANOVA) of Montepulciano samples at harvest time and after 7 and 15 days of spontaneous fermentation. Letters O, C and NT indicated organic, conventional and untreated farming management, respectively. For each yeast species detected and for each sampling time, the different letters (A, B) indicated significant differences between the samples (p < 0.05) using t-Test. (Table D) Viable cell count (CFU/ml) of Verdicchio samples (V) subjected to organic (VO), conventional (VC) and untreated (VNT) farming managements, at harvest time, after 7 and 15 days of spontaneous fermentation. (Table E) Viable cell count (CFU/ml) Montepulciano samples (M) subjected to organic (MO), conventional (MC) and untreated (MNT) farming managements, at harvest time and after 7 and 15 days of spontaneous fermentation.
(DOCX)
