# Supplementary material for: The impact of fungicide treatments on yeast biota of Verdicchio and Montepulciano grape varieties
Source: PLoS One. 2019 Jun 20;14(6):e0217385. doi: 10.1371/journal.pone.0217385 (PMC6586281; doi:10.1371/journal.pone.0217385)
Supplement: S1 File — (Table A) Analysis of variance (ANOVA) of Verdicchio and Montepulciano samples at harvest time. The significant differences were determined using t-Test, and the data were considered significant if the associated P values was <0.05. Data with different letters (A, B) within each row are significantly different. (Table B) Analysis of variance (ANOVA) of Verdicchio samples at harvest time and after 7 and 15 days of spontaneous fermentation. Letters O, C and NT indicated organic, conventional and untreated farming management, respectively. For each yeast species detected and for each sampling time, the different letters (A, B) indicated significant differences between the samples (p < 0.05) using t-Test. (Table C) Analysis of variance (ANOVA) of Montepulciano samples at harvest time and after 7 and 15 days of spontaneous fermentation. Letters O, C and NT indicated organic, conventional and untreated farming management, respectively. For each yeast species detected and for each sampling time, the different letters (A, B) indicated significant differences between the samples (p < 0.05) using t-Test. (Table D) Viable cell count (CFU/ml) of Verdicchio samples (V) subjected to organic (VO), conventional (VC) and untreated (VNT) farming managements, at harvest time, after 7 and 15 days of spontaneous fermentation. (Table E) Viable cell count (CFU/ml) Montepulciano samples (M) subjected to organic (MO), conventional (MC) and untreated (MNT) farming managements, at harvest time and after 7 and 15 days of spontaneous fermentation. (DOCX) [file pone.0217385.s001.docx]

**Supplemental materials**

**Table A**. Analysis of variance (ANOVA) of Verdicchio and Montepulciano samples at harvest time. The significant differences were determined using t-Test, and the data were considered significant if the associated P values was <0.05. Data with different letters (A, B) within each row are significantly different.

| **Yeast species** | **Grape varieties** | |
| --- | --- | --- |
|  | **Verdicchio** | **Montepulciano** |
| *A. pullulans* | A | A |
| *I. terricola* | A | A |
| *C. californica* | A | A |
| *Cryptococcus spp.* | A | A |
| *S. bacillaris* | A | A |
| *M. pulcherrima* | A | A |
| *H. uvarum* | B | A |
| *D. hansenii* | A | A |
| *P. fermentans* | nd* | nd |
| *P. sporocuriosa* | nd | nd |
| *P. membranifaciens* | nd | nd |
| *Rhodotorula spp.* | nd | nd |
| *Z. bailii* | nd | nd |
| *Z. meyerae* | nd | nd |
| *L. thermotolerans* | nd | nd |

*nd= t-Test not detected. In particular, *P. fermentans*, *P. sporocuriosa* and *P. membranifaciens* species were detected only in Verdicchio samples, while *Rhodotorula* spp., *Z. bailii*, *Z. meyerae* and *L. thermotolerans* species were detected only in Montepulciano samples.

**Table B.** Analysis of variance (ANOVA) of Verdicchio samples at harvest time and after 7 and 15 days of spontaneous fermentation. Letters O, C and NT indicated organic, conventional and untreated farming management, respectively. For each yeast species detected and for each sampling time, the different letters (A, B) indicated significant differences between the samples (*p* < 0.05) using t-Test.

| **Yeast species** | **Harvest** | | | **7 days** | | | **15 days** | | |
| --- | --- | --- | --- | --- | --- | --- | --- | --- | --- |
|  | **O** | **C** | **NT** | **O** | **C** | **NT** | **O** | **C** | **NT** |
| *A. pullulans* | AB | A | B | nd* | nd | nd | nd | nd | nd |
| *I. terricola* | A | A | A | A | A | A | B | A | AB |
| *C. californica* | B | B | A | B | B | A | A | B | AB |
| *Cryptococcus spp.* | A | B | AB | nd | nd | nd | nd | nd | nd |
| *S. bacillaris* | A | A | A | A | A | A | A | A | A |
| *M. pulcherrima* | B | B | A | AB | B | A | A | A | A |
| *H. uvarum* | A | A | A | A | A | A | A | A | A |
| *D. hansenii* | A | A | A | nd | nd | nd | nd | nd | nd |
| *P. fermentans* | B | B | A | B | B | A | A | A | A |
| *P. sporocuriosa* | A | A | A | nd | nd | nd | nd | nd | nd |
| *P. membranifaciens* | A | A | A | nd | nd | nd | nd | nd | nd |
| *T. delbrueckii* | nd | nd | nd | A | A | A | A | A | A |
| *C. diversa* | nd | nd | nd | A | A | A | nd | nd | nd |
| *P. kudriavzewii* | nd | nd | nd | nd | nd | nd | B | B | A |
| *W. anomalus* | nd | nd | nd | nd | nd | nd | A | A | A |
| *S. cerevisiae* | nd | nd | nd | nd | nd | nd | A | A | A |

*nd= not detected

**Table C.** Analysis of variance (ANOVA) of Montepulciano samples at harvest time and after 7 and 15 days of spontaneous fermentation. Letters O, C and NT indicated organic, conventional and untreated farming management, respectively. For each yeast species detected and for each sampling time, the different letters (A, B) indicated significant differences between the samples (*p* < 0.05) using t-Test.

| **Yeast species** | **Harvest** | | | **7 days** | | | **15 days** | | |
| --- | --- | --- | --- | --- | --- | --- | --- | --- | --- |
|  | **O** | **C** | **NT** | **O** | **C** | **NT** | **O** | **C** | **NT** |
| *A. pullulans* | AB | A | B | nd* | nd | nd | nd | nd | nd |
| *I. terricola* | A | A | A | A | B | AB | A | A | A |
| *C. californica* | A | A | A | A | A | A | A | A | A |
| *Cryptococcus spp.* | A | A | A | nd | nd | nd | nd | nd | nd |
| *S. bacillaris* | A | B | AB | A | A | A | A | B | B |
| *M. pulcherrima* | B | AB | A | A | A | A | nd | nd | nd |
| *H. uvarum* | B | B | A | A | A | B | A | A | A |
| *D. hansenii* | A | A | A | nd | nd | nd | A | A | A |
| *Rhodotorula spp.* | A | A | A | nd | nd | nd | nd | nd | nd |
| *Z. bailii* | A | A | A | A | A | A | A | A | A |
| *Z. meyerae* | A | A | A | nd | nd | nd | nd | nd | nd |
| *L. thermotolerans* | A | A | A | A | A | A | A | A | A |
| *P. fermentans* | nd | nd | nd | nd | nd | nd | A | A | A |
| *P. sporocuriosa* | nd | nd | nd | A | A | A | B | A | AB |

*nd= not detected

**Table D.** Viable cell count (CFU/ml) of Verdicchio samples (V) subjected to organic (VO), conventional (VC) and untreated (VNT) farming managements, at harvest time, after 7 and 15 days of spontaneous fermentation.

| **Samples** | **Sampling time** | **Yeast species (CFU/ml)** | | | | | | | | | | | | | | | |
| --- | --- | --- | --- | --- | --- | --- | --- | --- | --- | --- | --- | --- | --- | --- | --- | --- | --- |
|  |  | *A. pullulans* | *I. terricola* | *C. californica* | *Cryptococcus spp.* | *S. bacillaris* | *M. pulcherrima* | *H. uvarum* | *D. hansenii* | *P. fermentans* | *P. sporocuriosa* | *P. membranifaciens* | *T. delbrueckii* | *C. diversa* | *P. kudriavzewii* | *W. anomalus* | *S. cerevisiae* |
|  | Harvest | 1,10E+04 |  |  | 4,00E+03 | 1,00E+03 | 1,10E+04 |  |  |  |  |  |  |  |  |  |  |
| VO1 | 7 days |  |  |  |  |  |  | 3,70E+07 |  | 1,00E+05 |  |  |  |  |  |  |  |
|  | 15 days |  |  |  |  |  | 1,51E+05 | 2,67E+07 |  |  |  |  |  |  |  |  |  |
|  | Harvest | 2,20E+04 |  |  |  |  |  | 7,35E+04 |  |  |  | 8,00E+03 |  |  |  |  |  |
| VO2 | 7 days |  | 2,00E+04 | 8,00E+05 |  |  | 7,05E+05 | 5,78E+07 |  | 2,20E+05 |  |  |  |  |  |  |  |
|  | 15 days |  |  |  |  |  |  | 9,30E+05 |  | 3,07E+06 |  |  |  |  |  |  |  |
|  | Harvest | 8,00E+03 |  |  | 5,00E+03 |  |  |  |  |  |  |  |  |  |  |  |  |
| VO3 | 7 days |  |  |  |  |  | 1,00E+05 | 8,84E+07 |  |  |  |  |  |  |  |  |  |
|  | 15 days |  |  |  |  |  |  | 2,91E+07 |  |  |  |  |  |  |  |  |  |
|  | Harvest | 2,30E+04 |  |  | 4,00E+03 |  | 1,00E+03 |  |  |  |  |  |  |  |  |  |  |
| VO4 | 7 days |  |  |  |  |  | 1,00E+04 | 5,17E+07 |  |  |  |  |  |  |  |  |  |
|  | 15 days |  |  |  |  |  | 1,45E+06 | 2,96E+07 |  |  |  |  |  |  |  |  |  |
|  | Harvest |  |  |  | 1,40E+04 |  |  | 4,15E+04 |  |  |  | 7,00E+03 |  |  |  |  |  |
| VO5 | 7 days |  | 2,22E+06 |  |  |  |  | 7,87E+07 |  | 8,55E+05 |  |  |  |  |  |  |  |
|  | 15 days |  |  |  |  | 4,90E+04 |  |  |  |  |  |  |  |  |  |  |  |
|  | Harvest | 9,00E+03 | 1,00E+03 |  | 5,00E+03 | 1,00E+04 |  |  |  |  |  |  |  |  |  |  |  |
| VO6 | 7 days |  |  |  |  |  | 1,00E+04 | 7,83E+07 |  |  |  |  |  |  |  |  |  |
|  | 15 days |  |  | 1,31E+06 |  |  |  |  |  |  |  |  |  |  |  |  |  |
|  | Harvest | 8,00E+03 |  |  | 6,00E+03 | 2,00E+03 |  |  |  |  |  | 5,00E+03 |  |  |  |  |  |
| VO7 | 7 days |  |  | 8,00E+04 |  |  |  | 2,75E+07 |  | 7,50E+05 |  |  |  |  |  |  |  |
|  | 15 days |  |  | 6,35E+06 |  | 6,00E+04 |  | 2,10E+05 |  |  |  |  |  |  |  |  |  |
|  | Harvest | 1,00E+03 |  |  | 1,00E+03 |  |  |  |  |  |  |  |  |  |  |  |  |
| VO8 | 7 days |  | 5,00E+05 |  |  |  | 2,00E+04 | 5,79E+07 |  | 2,00E+04 |  |  |  |  |  |  |  |
|  | 15 days |  |  | 1,98E+06 |  |  |  | 1,07E+07 |  |  |  |  |  |  |  |  |  |
|  | Harvest | 4,65E+04 |  |  |  |  |  | 6,50E+04 |  |  |  |  |  |  |  |  |  |
| VO9 | 7 days |  | 1,10E+05 |  |  |  | 2,05E+05 | 8,42E+07 |  | 1,00E+04 |  |  | 2,00E+04 |  |  |  |  |
|  | 15 days |  |  | 8,22E+06 |  |  |  | 3,65E+07 |  |  |  |  |  |  |  |  |  |
|  | Harvest | 3,10E+04 |  |  | 6,00E+03 | 1,40E+04 |  | 4,00E+03 |  |  |  |  |  |  |  |  |  |
| VO10 | 7 days |  | 2,95E+06 |  |  |  |  | 5,81E+07 |  | 5,00E+05 |  |  | 1,00E+04 |  |  |  |  |
|  | 15 days |  |  | 2,89E+06 |  | 2,40E+05 | 2,00E+04 |  |  |  |  |  |  |  |  |  |  |
|  | Harvest | 6,10E+04 |  |  | 3,00E+03 | 4,00E+03 |  | 4,00E+03 | 2,00E+05 |  |  |  |  |  |  |  |  |
| VO11 | 7 days |  | 1,31E+06 |  |  |  | 7,85E+05 | 5,65E+07 |  |  |  |  |  |  |  |  |  |
|  | 15 days |  |  | 9,40E+06 |  |  | 8,35E+04 | 5,26E+07 |  | 1,50E+05 |  |  |  |  |  |  |  |
|  | Harvest | 1,20E+05 |  |  |  | 1,00E+05 |  | 1,41E+05 |  |  |  | 1,00E+03 |  |  |  |  |  |
| VO12 | 7 days |  | 1,00E+04 |  |  |  |  | 4,44E+07 |  |  |  |  |  |  |  |  |  |
|  | 15 days |  |  | 2,25E+04 |  |  | 7,25E+04 | 4,60E+05 |  |  |  |  |  |  |  |  |  |
|  | Harvest | 1,00E+04 | 8,00E+03 |  |  | 7,00E+03 |  | 3,60E+04 |  |  | 1,00E+04 | 1,00E+04 |  |  |  |  |  |
| VO13 | 7 days |  | 2,85E+05 | 1,00E+05 |  | 1,00E+05 |  | 2,19E+07 |  |  |  |  |  |  |  |  |  |
|  | 15 days |  |  | 2,42E+05 |  | 3,95E+04 |  |  |  |  |  |  |  |  |  |  |  |
|  | Harvest | 7,80E+04 |  |  | 7,00E+03 |  |  |  |  |  |  |  |  |  |  |  |  |
| VC1 | 7 days |  | 3,00E+04 |  |  |  |  | 3,21E+07 |  | 3,00E+05 |  |  |  |  |  |  |  |
|  | 15 days |  | 5,05E+06 |  |  |  |  | 2,95E+07 |  | 3,20E+05 |  |  |  |  |  |  | 1,66E+06 |
|  | Harvest | 2,70E+04 |  |  |  |  |  |  |  |  |  |  |  |  |  |  |  |
| VC2 | 7 days |  |  |  |  |  | 5,50E+04 | 9,51E+07 |  |  |  |  |  |  |  |  |  |
|  | 15 days |  |  |  |  |  |  | 1,15E+07 |  |  |  |  | 2,21E+06 |  |  | 8,41E+06 |  |
|  | Harvest | 5,35E+04 | 1,35E+04 |  |  |  |  | 1,47E+05 |  |  |  |  |  |  |  |  |  |
| VC3 | 7 days |  | 2,16E+06 |  |  |  | 9,00E+04 | 7,67E+07 |  |  |  |  |  |  |  |  |  |
|  | 15 days |  | 2,32E+06 |  |  |  |  | 1,66E+07 |  |  |  |  | 3,76E+06 |  |  |  |  |
|  | Harvest | 2,70E+04 |  |  |  |  |  | 3,10E+04 |  |  |  |  |  |  |  |  |  |
| VC4 | 7 days |  |  |  |  |  |  | 3,74E+07 |  |  |  |  |  |  |  |  |  |
|  | 15 days |  |  |  |  |  |  | 1,55E+07 |  |  |  |  |  |  | 1,31E+05 |  |  |
|  | Harvest | 3,70E+04 |  |  |  |  |  | 3,00E+04 |  |  |  |  |  |  |  |  |  |
| VC5 | 7 days |  |  |  |  |  |  | 4,53E+07 |  |  |  |  |  |  |  |  |  |
|  | 15 days |  | 3,16E+06 |  |  |  |  | 2,11E+07 |  |  |  |  | 5,00E+04 |  |  |  |  |
|  | Harvest | 5,00E+03 |  |  | 1,00E+03 |  |  |  |  |  |  |  |  |  |  |  |  |
| VC6 | 7 days |  |  |  |  |  |  | 6,07E+07 |  | 1,30E+05 |  |  |  | 1,00E+04 |  |  |  |
|  | 15 days |  |  |  |  |  |  | 8,76E+06 |  |  |  |  | 1,90E+05 |  |  | 1,87E+07 |  |
|  | Harvest | 3,00E+03 |  |  |  |  |  |  |  |  |  |  |  |  |  |  |  |
| VC7 | 7 days |  | 1,00E+05 |  |  |  |  | 5,58E+07 |  |  |  |  |  |  |  |  |  |
|  | 15 days |  | 8,00E+05 |  |  |  |  | 9,36E+06 |  |  |  |  |  |  |  |  |  |
|  | Harvest | 2,15E+04 |  |  |  | 3,26E+05 |  | 4,50E+04 |  |  |  |  |  |  |  |  |  |
| VC8 | 7 days |  |  |  |  | 1,14E+07 |  | 9,60E+06 |  |  |  |  |  |  |  |  |  |
|  | 15 days |  |  |  |  | 1,00E+04 |  | 1,00E+03 |  |  |  |  |  |  |  |  |  |
|  | Harvest | 2,05E+04 |  |  | 5,50E+04 | 8,51E+05 |  | 1,58E+05 |  |  |  |  |  |  |  |  |  |
| VC9 | 7 days |  |  |  |  |  |  | 4,32E+07 |  | 1,00E+05 |  |  |  |  |  |  |  |
|  | 15 days |  |  |  |  |  |  |  |  |  |  |  |  |  |  |  |  |
|  | Harvest | 1,24E+05 |  |  |  |  |  |  |  |  |  |  |  |  |  |  |  |
| VC10 | 7 days |  |  |  |  |  | 1,70E+05 | 7,37E+07 |  | 2,00E+04 |  |  |  |  |  |  |  |
|  | 15 days |  |  |  |  |  | 1,00E+04 | 2,53E+07 |  | 2,40E+05 |  |  | 1,00E+04 |  |  | 4,79E+06 |  |
|  | Harvest | 3,55E+04 |  | 1,55E+04 |  | 6,70E+04 | 2,20E+04 | 2,45E+05 |  | 1,45E+04 |  |  |  |  |  |  |  |
| VNT1 | 7 days |  |  | 3,03E+06 |  |  | 7,00E+04 | 9,04E+07 |  | 4,38E+06 |  |  |  |  |  |  |  |
|  | 15 days |  |  |  |  | 1,12E+06 |  |  |  |  |  |  |  |  | 4,65E+04 |  |  |
|  | Harvest | 1,10E+05 |  | 8,00E+04 |  | 1,77E+06 | 8,90E+05 | 1,37E+06 |  | 5,00E+04 |  |  |  |  |  |  |  |
| VNT2 | 7 days |  |  | 3,05E+05 |  |  | 8,65E+05 | 6,13E+07 |  | 5,00E+04 |  |  |  |  |  |  |  |
|  | 15 days |  |  |  |  |  |  |  |  |  |  |  |  |  | 1,00E+03 |  |  |

**Table E.** Viable cell count (CFU/ml) Montepulciano samples (M) subjected to organic (MO), conventional (MC) and untreated (MNT) farming managements, at harvest time and after 7 and 15 days of spontaneous fermentation.

| **Samples** | **Sampling time** | **Yeast species (CFU/ml)** | | | | | | | | | | | | | |
| --- | --- | --- | --- | --- | --- | --- | --- | --- | --- | --- | --- | --- | --- | --- | --- |
|  |  | *A. pullulans* | *I. terricola* | *C. californica* | *Cryptococcus spp.* | *S. bacillaris* | *M. pulcherrima* | *H. uvarum* | *D. hansenii* | *P. fermentans* | *P. sporocuriosa* | *Z. bailii* | *L. thermotolerans* | *Rhodotorula spp.* | *Z. meyerae* |
|  | Harvest | 5,00E+03 |  |  |  |  |  |  |  |  |  |  |  | 1,00E+03 |  |
| MO1 | 7 days |  |  |  |  |  | 5,00E+05 | 1,39E+08 |  |  |  |  |  |  |  |
|  | 15 days |  |  |  |  |  |  |  |  |  |  |  |  |  |  |
|  | Harvest | 1,00E+03 | 1,00E+04 |  |  |  |  |  |  |  |  |  |  |  |  |
| MO2 | 7 days |  | 4,20E+05 |  |  |  |  | 5,51E+07 |  |  |  |  |  |  |  |
|  | 15 days |  | 1,59E+07 |  |  | 4,96E+07 |  |  |  |  |  |  |  |  |  |
|  | Harvest | 1,00E+04 |  |  |  | 6,40E+05 |  | 2,47E+05 |  |  |  |  |  |  |  |
| MO3 | 7 days |  |  |  |  | 6,20E+05 |  |  |  |  |  |  |  |  |  |
|  | 15 days |  |  |  |  |  |  |  |  |  |  | 7,60E+04 |  |  |  |
|  | Harvest | 6,55E+04 |  |  | 1,00E+03 | 1,00E+03 |  | 1,55E+04 |  |  |  |  |  |  |  |
| MO4 | 7 days |  | 1,00E+06 | 1,00E+06 |  | 6,50E+06 |  | 1,00E+05 |  |  |  |  |  |  |  |
|  | 15 days |  |  | 1,27E+05 |  | 3,00E+04 |  | 1,00E+04 |  |  |  |  |  |  |  |
|  | Harvest | 1,00E+03 |  |  | 1,73E+05 |  |  | 6,70E+04 |  |  |  |  |  |  | 2,20E+04 |
| MO5 | 7 days |  | 7,50E+05 |  |  | 4,50E+06 |  | 1,41E+07 |  |  |  |  |  |  |  |
|  | 15 days |  | 3,00E+05 |  |  | 8,90E+06 |  |  | 2,00E+05 |  |  |  |  |  |  |
|  | Harvest | 7,00E+04 | 2,43E+06 |  |  | 4,40E+05 |  | 1,75E+06 |  |  |  |  |  |  | 9,00E+04 |
| MO6 | 7 days |  | 2,00E+06 | 1,80E+06 |  | 1,11E+07 |  | 1,87E+07 |  |  |  |  |  |  |  |
|  | 15 days |  |  |  |  |  |  |  | 3,34E+05 |  |  |  |  |  |  |
|  | Harvest | 4,30E+04 |  |  |  | 1,00E+04 |  | 3,55E+04 |  |  |  |  |  |  |  |
| MO7 | 7 days |  |  | 4,25E+05 |  |  |  | 5,99E+07 |  |  | 3,15E+05 |  |  |  |  |
|  | 15 days |  | 2,00E+05 | 1,13E+07 |  |  |  | 1,22E+07 |  | 8,00E+04 |  |  |  |  |  |
|  | Harvest |  |  |  |  | 2,80E+05 |  | 6,10E+05 |  |  |  |  |  |  | 3,00E+04 |
| MO8 | 7 days |  |  | 1,14E+07 |  | 5,20E+06 |  | 1,58E+07 |  |  |  |  |  |  |  |
|  | 15 days |  |  |  |  | 1,23E+07 |  |  | 6,20E+05 |  |  |  |  |  |  |
|  | Harvest |  | 8,50E+03 |  |  | 3,20E+05 |  | 1,16E+06 |  |  |  |  |  |  | 7,00E+03 |
| MO9 | 7 days |  | 1,21E+07 |  |  | 3,20E+06 |  | 9,50E+06 |  |  |  |  |  |  |  |
|  | 15 days |  |  | 4,00E+05 |  | 2,00E+05 |  |  |  |  |  |  |  |  |  |
|  | Harvest |  |  | 7,00E+04 |  | 3,00E+05 |  | 9,30E+05 |  |  |  |  |  |  |  |
| MO10 | 7 days |  | 1,23E+07 |  |  | 1,70E+06 |  | 2,45E+07 |  |  |  |  |  |  |  |
|  | 15 days |  |  |  |  | 1,90E+05 |  |  |  |  |  | 5,00E+04 |  |  |  |
|  | Harvest | 8,05E+04 |  |  |  |  |  |  |  |  |  |  |  | 1,40E+04 |  |
| MC1 | 7 days |  | 1,70E+05 |  |  |  |  | 7,42E+07 |  |  |  |  |  |  |  |
|  | 15 days |  |  |  |  |  |  |  |  |  |  |  |  |  |  |
|  | Harvest | 2,75E+04 |  |  | 2,00E+03 |  | 2,00E+03 | 3,25E+04 |  |  |  |  |  | 2,00E+03 |  |
| MC2 | 7 days |  | 2,00E+04 |  |  |  | 4,88E+06 | 1,30E+08 |  |  |  |  |  |  |  |
|  | 15 days |  |  |  |  |  |  |  |  |  | 2,00E+03 |  |  |  |  |
|  | Harvest | 7,40E+04 |  |  | 2,00E+03 |  | 3,00E+03 | 1,15E+04 |  |  |  |  |  |  |  |
| MC3 | 7 days |  | 1,00E+04 |  |  |  | 1,44E+06 | 6,92E+07 |  |  |  |  |  |  |  |
|  | 15 days |  |  |  |  |  |  |  |  |  | 5,00E+03 |  |  |  |  |
|  | Harvest | 3,65E+04 |  |  |  |  |  | 3,00E+03 |  |  |  |  |  | 2,00E+03 |  |
| MC4 | 7 days |  | 1,00E+04 |  |  |  | 1,41E+06 | 1,28E+08 |  |  |  |  |  |  |  |
|  | 15 days |  |  | 3,44E+07 |  | 4,10E+06 |  | 3,51E+07 |  |  |  |  |  |  |  |
|  | Harvest | 9,55E+04 | 2,00E+03 |  | 2,00E+03 |  |  | 3,00E+03 |  |  |  |  | 1,25E+05 |  |  |
| MC5 | 7 days |  |  |  |  |  | 3,00E+04 | 2,91E+07 |  |  |  |  | 5,18E+07 |  |  |
|  | 15 days |  |  |  |  |  |  |  |  |  |  |  | 8,14E+07 |  |  |
|  | Harvest |  |  |  |  |  | 2,96E+05 | 4,22E+06 |  |  |  |  |  |  | 1,00E+04 |
| MC6 | 7 days |  |  | 1,00E+04 |  | 4,10E+05 |  |  |  |  |  |  |  |  |  |
|  | 15 days |  |  |  |  |  |  |  |  |  |  | 1,83E+05 |  |  |  |
|  | Harvest | 5,40E+04 |  |  | 4,00E+03 |  |  | 3,70E+04 |  |  |  |  |  | 3,00E+03 |  |
| MC7 | 7 days |  |  |  |  |  | 3,40E+05 | 6,61E+07 |  |  |  |  |  |  |  |
|  | 15 days |  |  |  |  |  |  | 1,00E+05 |  |  | 1,19E+06 |  |  |  |  |
|  | Harvest | 1,60E+04 |  |  |  |  |  | 1,80E+04 |  |  |  |  |  |  |  |
| MC8 | 7 days |  |  |  |  |  | 2,00E+04 | 1,03E+08 |  |  |  |  |  |  |  |
|  | 15 days |  |  |  |  |  |  | 8,60E+07 |  |  |  |  |  |  |  |
|  | Harvest |  | 2,00E+03 |  | 4,00E+03 |  | 1,40E+04 | 2,65E+06 |  |  |  |  |  |  | 2,00E+03 |
| MC9 | 7 days |  |  | 1,38E+07 |  | 2,80E+06 |  | 2,09E+07 |  |  |  |  |  |  |  |
|  | 15 days |  |  | 1,22E+07 |  | 7,50E+06 |  |  |  |  |  |  |  |  |  |
|  | Harvest | 5,25E+04 |  |  |  |  |  | 1,50E+05 | 1,29E+05 |  |  |  |  |  |  |
| MC10 | 7 days |  |  |  |  |  |  | 6,88E+07 |  |  |  |  |  |  |  |
|  | 15 days |  |  |  |  | 3,70E+05 |  |  | 7,20E+07 |  |  |  |  |  |  |
|  | Harvest | 1,20E+05 | 4,00E+03 |  |  |  | 2,00E+03 | 1,01E+06 |  |  |  |  |  |  |  |
| MNT1 | 7 days |  |  |  |  |  |  |  |  |  |  |  |  |  |  |
|  | 15 days |  |  |  |  |  |  |  |  |  |  |  |  |  |  |
|  | Harvest | 2,10E+04 |  |  |  |  | 8,00E+03 | 1,52E+05 |  |  |  |  |  |  |  |
| MNT2 | 7 days |  |  |  |  |  |  |  |  |  |  |  |  |  |  |
|  | 15 days |  |  |  |  |  |  |  |  |  |  |  |  |  |  |
|  | Harvest |  |  |  |  | 2,00E+04 | 4,00E+04 | 7,20E+05 |  |  |  |  |  |  | 1,70E+05 |
| MNT3 | 7 days |  |  | 1,72E+06 |  | 2,20E+05 |  | 2,80E+05 |  |  |  |  |  |  |  |
|  | 15 days |  |  | 6,60E+05 |  |  |  |  |  |  |  | 8,50E+04 |  |  |  |
|  | Harvest |  | 7,50E+05 |  |  | 2,80E+05 | 1,40E+05 | 1,70E+06 |  |  |  | 9,50E+03 |  |  |  |
| MNT4 | 7 days |  |  |  |  |  |  |  |  |  |  | 6,00E+04 |  |  |  |
|  | 15 days |  |  |  |  |  |  |  |  |  |  | 2,90E+05 |  |  |  |
|  | Harvest | 1,70E+05 |  |  |  | 3,30E+04 | 5,00E+03 | 4,50E+05 |  |  |  |  |  | 1,00E+03 |  |
| MNT5 | 7 days |  |  |  |  | 6,00E+04 |  |  |  |  |  |  |  |  |  |
|  | 15 days |  |  |  |  |  |  |  |  |  |  |  |  |  |  |
